# Eocene and modern entomofauna differ—a Cretaceous‐like larva in Rovno amber

**DOI:** 10.1111/1744-7917.13410

**Published:** 2024-07-15

**Authors:** Joachim T. Haug, Simon Linhart, Viktor Baranov, Carolin Haug

**Affiliations:** ^1^ Faculty of Biology, Ludwig‐Maximilians‐Universität München (LMU Munich) Planegg‐Martinsried Germany; ^2^ GeoBio‐Center at LMU München Germany; ^3^ Estación Biológica de Doñana‐CSIC Seville Spain

## Abstract

We report a 35 million‐year‐old lacewing larva from Ukrainian amber. This insect larva has a morphology up to now only known from 100 million‐year‐old amber. Therefore, this morphology survived more than 60 million years longer than previously assumed. Our find contradicts the common notion that the fauna 35 million years ago was already very modern.

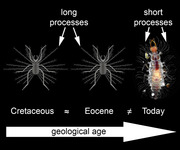

Dear Editor,

The Eocene (ca. 56–34 million years ago) has become an important time to study due to the global warming at the time (e.g., Inglis *et al.*, 2020; Agterhuis *et al.*, 2022; Tierney *et al.*, 2022; Setty *et al.*, 2023), hence providing a comparison to modern climate change. The Eocene is also well known for spectacular fossils of the group Insecta, with flies, beetles, moths, and all their kin, not least due to exceptional preservation in different types of ambers, most well known the Baltic amber (Janzen, 2002; Weitschat & Wichard, 2002; Gröhn, 2015). The detailed preservation of the often tiny animals from Baltic and other Eocene ambers—such as Oise amber (France; Nel *et al.*, 2004; Brasero *et al.*, 2009; Kirejtshuk & Nel, 2013), Saxonian or Bitterfeld amber (Germany; Wichard, 2013; Bukejs *et al.*, 2016; Dunlop *et al.*, 2018), Fushun amber (China; Wang *et al.*, 2014, 2011; Zhang *et al.*, 2016), Cambay amber (India; Rust *et al.*, 2010; Engel *et al.*, 2013; Stebner *et al.*, 2017) or Rovno amber (Ukraine; Perkovsky *et al.*, 2003; Bukejs *et al.*, 2020; Lyubarsky *et al.*, 2023)—has provided a thorough basis for comparisons with the modern fauna. The overall impression seems to be that the Eocene entomofauna was already overall modern (e.g., Cockerell, 1920). This impression is supported by quite differing Mesozoic and Cenozoic entomofaunas, with only few surviving morphotypes from the one to the other. The pattern of very modern‐appearing morphotypes in post‐Cretaceous faunas with barely any survivor from the Mesozoic has, for example, been observed in cockroaches (Gorochov, 2007; Vršanský *et al.*, 2011, 2012, 2013; Greenwalt & Vidlička, 2015) or non‐biting midges (Seredszus & Wichard, 2011). In the latter case, more than 90% of non‐biting midge species known from Eocene Oise and Baltic ambers have been interpreted as representatives of genera with modern‐day species (e.g., Seredszus & Wichard, 2002, 2007; Doitteau & Nel, 2007; Wichard *et al.*, 2009; Baranov *et al.*, 2015). Yet, a single morphotype seems indeed to have crossed the Cretaceous‐Cenozoic boundary (66 million years ago) disappearing toward the modern fauna (Baranov *et al.*, 2019a), already indicating that the case is likely less simple.

Quantitative analysis of lacewing larvae and their relatives, Neuropterida, has demonstrated that the Eocene fauna was intermediate in diversity between the more diverse Cretaceous and the less diverse modern fauna for some of the neuropteridan lineages (Haug *et al.*, 2020a, b, 2022a–c, 2023a; Hassenbach *et al.*, 2023), indicating that at least some morphotypes have crossed the boundary into the Cenozoic just to go extinct afterward. In addition, some morphologies seem to have been present only in the Eocene, not seen before and after (MacLeod, 1970; Haug *et al.*, 2023a, 2022b, 2023a; Mengel *et al.*, 2023), which is also observed in some other lineages (Baranov *et al.*, 2019b). Still, many of the extreme morphologies seen in Cretaceous neuropteridan larvae (Pérez‐de la Fuente *et al.*, 2012, 2016, 2016, 2018, 2019; Badano *et al.*, 2018, 2021, 2021; Haug *et al.*, 2019a, 2019b, b, 2020a, 2020b, b, 2021, 2022d, 2023a, 2023b; Zippel *et al.*, 2021) seem to have disappeared at the K‐Pg boundary or before.

Here we report a new lacewing larva preserved in Eocene (ca. 35 myo) Rovno amber, Ukraine (Fig. 1). The larva shows many characters known from Cretaceous specimens, but absent in other Eocene larvae and also extant ones. Hence, the specimen represents another case of a morphotype surviving into the Cenozoic. For details on the origin of the specimen, the documentation methods, and the morphology, see Supplementary material.

**Fig. 1:**
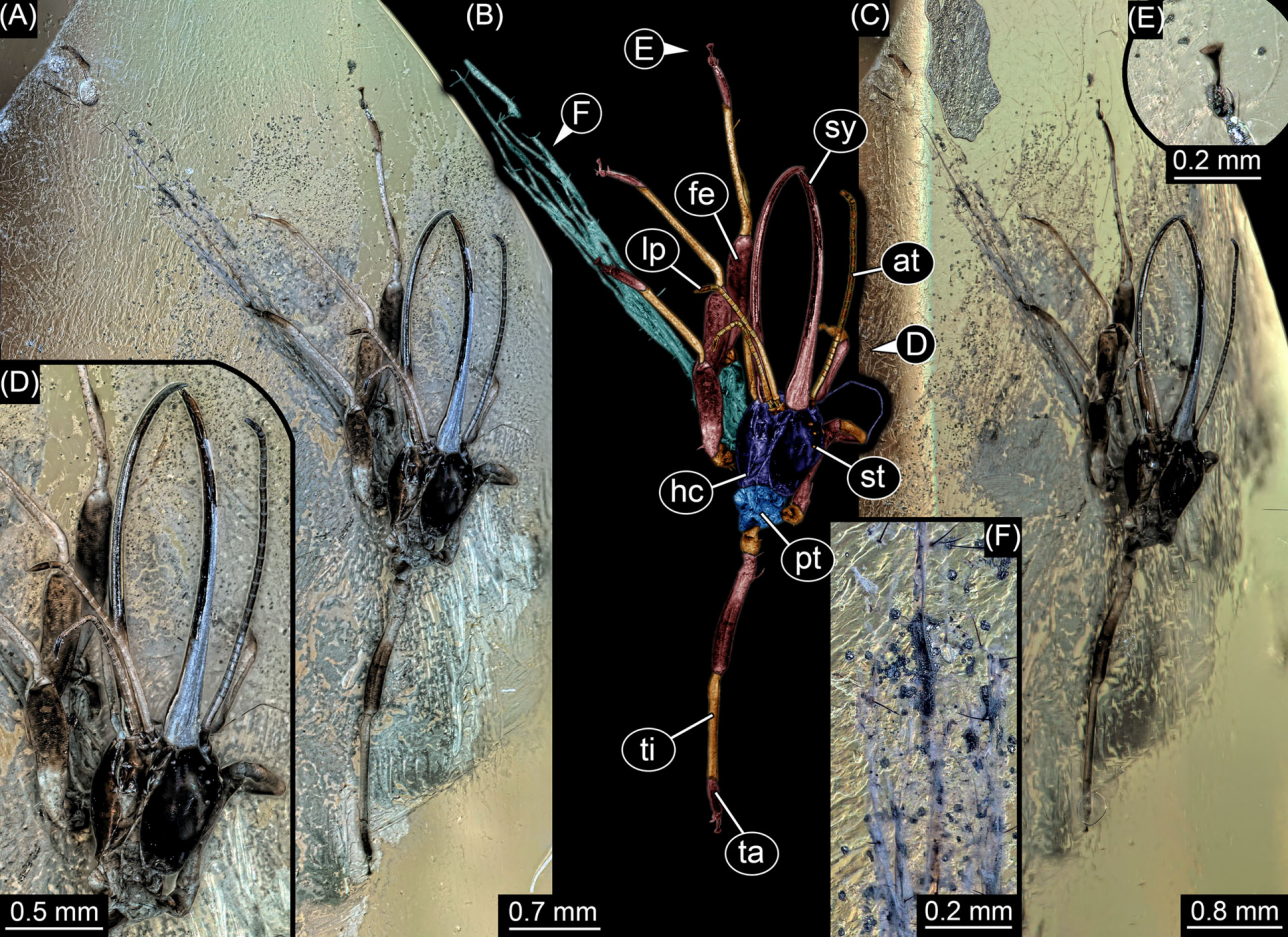
New aphidlion preserved in Eocene Rovno amber, PED 3530. (A) Overview. (B) Colour‐marked version of A. (C) Slightly tilted view. (D) Close‐up on head region. (E) Close‐up on distal part of leg with claws and empodium. (F) Close‐up on dorsal processes. Abbreviations: at = antenna; fe = femur; hc = head capsule; lp = labial palp; pt = prothorax; st = stemma; sy = stylet; ta = tarsus; ti = tibia.

The new fossil is partly deformed and twisted. Still, major features can be observed: the stylet of the larva is inward curved, elongate (more than twice the length of the head capsule), and lacks teeth. The distal parts of the legs each bear a trumpet‐shaped empodium. This combination of characters is well known for aphidlions, that is, extant larvae of the groups Chrysopidae (green lacewings) and Hemerobiidae (brown lacewings) and their fossil relatives. The presence of long setose dorsal processes is especially known in Cretaceous relatives of green lacewing larvae (Chrysopoidea), which are using these to attach a camouflaging cloak (Wang *et al.*, 2016). While the body is twisted, the dorsal processes are well preserved with details and their appearance has presumably not been affected by preservation.

In detail, the processes seen in the new fossil are most similar to those of a larva reported from Cretaceous Álava amber (Spain, ca. 110–100 million years old; Pérez‐de la Fuente *et al.*, 2012; Fig. 2D) as in both the setae arising from the long processes are rather short. In other larvae with long processes from Lebanon (ca. 130 million years old; Pérez‐de la Fuente *et al.*, 2018, 2019; Fig. 2E, F) and Myanmar amber (ca. 100 million years old; Fig. 2A–C; Wang *et al.*, 2016; Haug *et al.*, 2022d), the setae are longer in comparison to the processes.

**Fig. 2:**
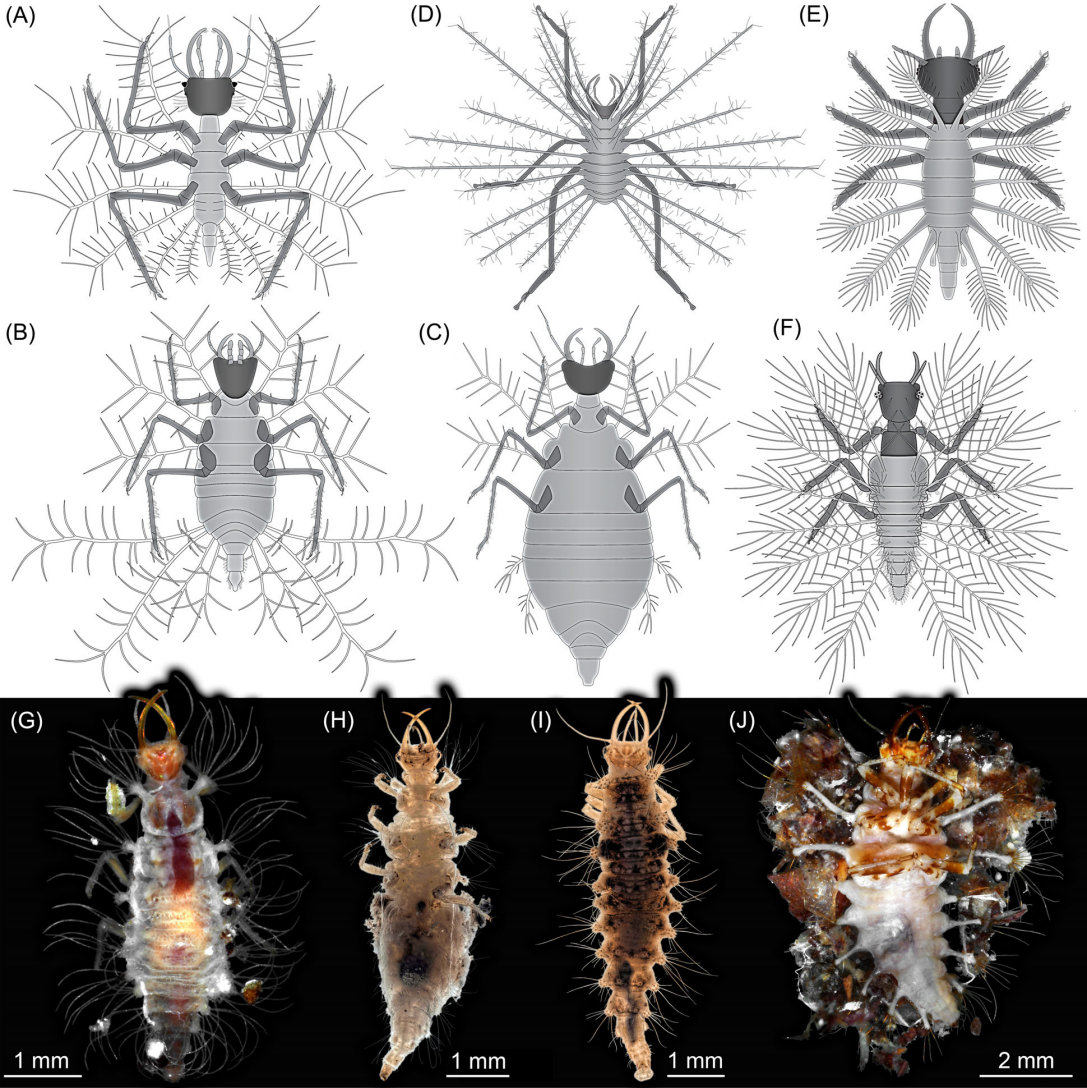
Cretaceous and modern aphidlions with dorsal processes. (A–C) Larvae from about 100 million years old Kachin amber, Myanmar; from Haug *et al.* (2022d). (A) Type 1. (B) Type 2. (C) Type 3. (D) Larva from 110–100 million years old Spanish amber; based on Pérez‐de la Fuente *et al.* (2012, 2016). (E–F) Larvae from about 130 million years old Lebanon amber. (E) Based on Pérez‐de la Fuente *et al.* (2019). (F) Based on Pérez‐de la Fuente *et al.* (2018). (G–J) Modern‐day larvae. (G) ZMH 62888. (H) ESMNS_67, flipped horizontally. (I) ESMNS_017. (J) ZMH 62915, possibly *Leucochrysa* due to the relatively long dorsal processes.

Modern‐day aphidlions differ from the here reported larva (and many Cretaceous larvae). In modern larvae, even “long” stylets are not more than twice as long as the head capsule (Adams, 1959 p. 22 fig. 4b). Stylets of this length, or even longer, are only known in Cretaceous larvae (Haug *et al.*, 2021 p. 3 fig. 2A; Haug *et al.*, 2022d pp. 9, 10, 20, 35 figs. 7, 9A, 23, 41).

Also in modern larvae, if they possess dorsal processes, these are often either short (Brauer, 1851 pl. II fig. 13; Froggatt, 1907 p. 65 fig. 40; Smith, 1926 p. 51 fig. 2; Principi, 1954 p. 369 fig. VI; Adams, 1959 p. 22 fig. 4a,c; Díaz‐Aranda & Monserrat, 1996 p. 92 fig. 12a; Tauber *et al.*, 1998 p. 610 fig. 4; Tauber, 2003 fig. 478 fig. 6) (Fig. 2G–I) or appear as simple strong setae without further structures arising from them (only in stage 1 larvae; Brauer, 1867 pl IXA; Monserrat, 1982 p. 114 fig. 2; Monserrat, 2008 p. 178 fig. 2b).

Among the modern larvae, the longest processes are known in the group *Leucochrysa* (Fig. 2J). Yet, also these strongly differ from the processes in the Cretaceous larvae, as the processes in the modern ones are much more massive and bear setae only distally (Mantoanelli *et al.*, 2011).

The apparent loss of specific characters of Cretaceous lacewing larvae indicates the loss of the ecological roles coupled to these morphologies in the later fauna (Haug *et al.*, 2023a). It seems that in few cases, these roles might have been taking over by larvae of other lineages (Braig *et al.*, 2023; Haug *et al.*, 2023a), but for many, it appears that they have not been substituted. We can only assume that these ecological roles were, for example, tied to now also extinct prey items (Haug *et al.*, 2023b), but the details so far remain unclear.

One other aspect in the new fossil seems more similar to modern‐day aphidlions. Labial palps of aphidlions generally have three elements, yet in some extant larvae, they appear to consist of more elements (Principi, 1947 p. 138 fig. IV; Principi, 1956 p. 340 fig. XIV; Toschi, 1965 p. 404 fig. 10; Tauber, 1974 p. 1140 fig. 12; Tsukaguchi, 1979 p. 361 fig. 4; Tauber, 2003 p. 477 fig. 5), although these may represent ornaments of the cuticle of a single element. This aspect appears similar in the new fossil; the palp appears to have more than three elements. However, there is in fact a single Cretaceous specimen that also shows indications of the subdivision (or ornament?) of the labial palp elements (Haug *et al.*, 2022d p. 35 fig. 41) and the specimen is indeed similar to the new larva concerning the relative stylet length. Yet, it lacks dorsal processes. The subdivision is therefore more common in the modern larvae but was already present in at least one Cretaceous species. The combination of this subdivision with the presence of the dorsal processes seems to be only present in the new larva.

Overall, the specimen is not a one‐to‐one similarity to a specific Cretaceous morphotype; for example, the rather long stylets are also well known in Cretaceous larvae, yet not necessarily in the ones with the long dorsal processes (Haug *et al.*, 2021 p. 3 fig. 2A; Haug *et al.*, 2022d p. 35 fig. 41). Only two specimens (Haug *et al.*, 2022d p. 13 fig. 13, p. 17 fig. 18) have dorsal processes and slightly longer stylets but not as long as in the new fossil. Still, both aspects, the long stylets and long dorsal processes, are known in this way in Cretaceous larvae and not in modern ones, and now in the new Eocene larva. One could argue that this morphology re‐evolved convergently in the Eocene after it became extinct at the end of the Cretaceous. Yet, given the degree of similarity, this suggestion seems less parsimonious than assuming that the new larva is a representative of Chrysopoidea and that some of the morphologies so far considered extinct after the end‐Cretaceous have in fact survived into the Eocene.

There are only few other known aphidlions in the Eocene, seven in total, all from Baltic amber. Three of these are larvae of brown lacewings (Hemerobiidae; Makarkin *et al.*, 2012; Haug *et al.*, 2022d p. 42 fig. 50; Haug *et al.*, 2023a) and lack dorsal camouflaging structures. The other four are larvae of green lacewings (Chrysopidae; Weitschat, 2009 p. 254 fig. 45; Haug *et al.*, 2022d pp. 40, 41 figs. 48C, 49A, C). All of these have dorsal structures for camouflaging but resembling those of modern types in being rather short.

The presence of an older morphotype of an aphidlion in Rovno amber and modern ones in Baltic amber also further underpins that these two faunas are not entirely similar (Perkovsky *et al.*, 2007; Mänd *et al.*, 2018; Perkovsky, 2018; Dunlop *et al.*, 2019; but see Szwedo & Sontag, 2013). The new fossil is also a qualitative support for the notion that the Eocene entomofauna still contained morphologies, and coupled to this ecologies, from the Cretaceous fauna and was therefore more intermediary. So far, such an intermediary condition could be shown quantitatively on other neuropteridan larvae, for silky lacewings and snakeflies (Haug *et al.*, 2020a,b, 2022a–2022c, 2020b, 2023a, 2023b; Hassenbach *et al.*, 2023). In addition, also special morphologies only known from the Eocene (MacLeod, 1970; Haug *et al.*, 2023a, 2022b; Mengel *et al.*, 2023) emphasise that the entomofauna at the time was not yet a modern‐day one.

## Supporting information



Details on the origin new aphidlion PED 3530 preserved in Eocene Rovno amber, the documentation methods applied in this study, and the morphology of the specimen.
