# Supplementary material for: Eocene and modern entomofauna differ—a Cretaceous‐like larva in Rovno amber
Source: Insect Sci. 2024 Jul 15;32(2):712–8. doi: 10.1111/1744-7917.13410 (PMC11976695; doi:10.1111/1744-7917.13410)
Supplement: Supplementary file 1 — Details on the origin new aphidlion PED 3530 preserved in Eocene Rovno amber, the documentation methods applied in this study, and the morphology of the specimen. [file INS-32-712-s001.docx]

**Supplementary material to “Eocene and modern entomofauna differ – a Cretaceous-like larva in Rovno amber”**

***Material and methods***

In the centre of this study is a single amber piece commercially bought from the trader Jonas Damzen (www.amberinclusions.eu). The piece is according to the source from Rovno amber, Ukraine. Due to the unusual nature of the larva, the piece was inspected with FTIR spectroscopy kindly provided by the company Bruker Optics. The piece showed a high similarity to other amber pieces from Ukraine, but clear differences to the most common type of Cretaceous amber, Kachin amber from Myanmar, and even more differences to younger resins such as copal or extant resin. Overall, the new piece indeed seems to originate from Ukraine. The specimen is now deposited in the Palaeo-Evo-Devo Research Group Collection of Arthropods, Ludwig-Maximilians-Universität München (LMU Munich), Germany under repository number PED 3530.

The specimen was documented on a Keyence VHX-6000 digital microscope. Various imaging methods were employed (fusion of stacks, merging of panoramas, HDR; Haug *et al.* 2019a).

Extant aphidlion larvae for comparison came from the collections of the Staatliches Museum für Naturkunde Stuttgart (SMNS) and the Leibniz-Institut zur Analyse des Biodiversitätswandels (LIB), formerly Centrum für Naturkunde (CeNak), Hamburg (ZMH). Specimens are stored under the repository numbers ZMH 62888 and ZMH 62915; the two SMNS specimens only received internal working numbers (ESMNS_67, ESMNS_017).

***Description of the new specimen PED 3530***

*General:* Small lacewing larva, apparently an exuvia, folded (Fig. 1A–C). Body with head and trunk region, head folded upwards-backwards, covering most of the trunk region. Only accessible from one side due to disturbances in the amber piece (Fig. 1A, C).

*Head:* Head segments forming a distinct head capsule. Head capsule about as long as wide, tapering posteriorly. All appendages arising strongly anteriorly (prognathous). Ocular segment recognisable by its main structures, simple eyes (stemmata); at least three, possibly five on one side (Fig. 1D). Post-ocular segment 1 recognisable by its pair of appendages (antennae); antenna slender, longer than head capsule, more than 2x; proximal region difficult to discern, distally elongate flagellum, subdivided into at least 28 elements; very proximal region of flagellum with a longer element, longer than wide, more than 3x; further distal 7 elements about as long as wide; even further distal elements longer than wide, 1.5x–2x. Terminal element distally rounded (Fig. 1D). No external structures of post-ocular segment 2 (intercalary segment). Post-ocular segment 3 and 4 recognisable by their pairs of appendages (mandibles and maxillae); each mandible and maxilla forming distinct compound structure (stylet); stylet elongate, curved inwards, tapering distally; longer than head capsule, about 2.5x (Fig. 1D). Post-ocular segment 5 recognisable by its appendages (labium); only distal parts (palps) apparent; palp shorter than antenna, but longer than head capsule, about 1.5x; palp with three major regions (interpreted as original three elements); proximal element stout, only partly visible; middle element elongate, making up major part of the palp, further subdivided into about 15 subunits; proximal region (about one quarter) not or only vaguely subdivided, longer than wide, about 5x; further distal part subdivided into 13 or 14 subunits, each subunit longer than wide, 1.5–2x; distal element slightly bellied proximally, distally tapering cone-shaped; longer than subunits, about 1.5x (Fig. 1D).

*Trunk:* Trunk region mostly concealed by head, appears strongly crumpled and folded, only protruding structures apparent. Ventrally locomotory appendages (legs) well apparent, at least five of them indicating post-ocular segments 6–8 (trunk segments 1–3, prothorax, mesothorax, metathorax); unclear which of these legs arises from which of the segments (Fig. 1A–C). Leg elongate, longer than antenna, with five major elements. Proximal element, coxa, stout, about as long as wide, with ample arthrodial membrane surrounding it. Element 2, trochanter, also about as long as wide, globular in appearance. Element 3, femur, elongate, weakly s-shaped, about as wide as coxa and trochanter, but much longer than wide, about 8x; distally tapering. In the distal two thirds, surface with a distinct reticulate pattern (Fig. 1D). Element 4, tibia, more slender, almost straight, only proximally with a weak bend and slightly widening, only about 0.5x the width of the femur, also slightly shorter than femur, longer than wide, about 11x; in some areas also indications of reticulate pattern. Distal element, tarsus, about as wide as tibia, but significantly shorter, still longer than wide, slightly more than 3x, distally slightly tapering, with two setae arising from the tapering region; in some areas also indications of reticulate pattern; distally with a pair of rather small hook-shaped claws and a large trumpet-shaped empodium (Fig. 1E). Claws about as long as tarsus wide, empodium much longer than claws, about 0.5x the length of the tarsus (Fig. 1E).

Additional structures arising from the trunk, presumably dorsally. At least five rod-like processes; longer than legs (Fig. 1B). Diameter slightly less than tibia and tarsus. Bearing numerous short and simple setae (Fig. 1F), slightly longer and stronger than the setae on the tarsus; longer towards the distal region of the process.
